# Lane and Road Marker Semantic Video Segmentation Using Mask Cropping and Optical Flow Estimation

**DOI:** 10.3390/s21217156

**Published:** 2021-10-28

**Authors:** Guansheng Xing, Ziming Zhu

**Affiliations:** School of Automation and Electronic Engineering, Qingdao University of Science and Technology, Qingdao 266061, China; xinggs@qust.edu.cn

**Keywords:** lane and road marker segmentation, mask cropping, optical flow estimation, semantic video segmentation, temporal consistency

## Abstract

Lane and road marker segmentation is crucial in autonomous driving, and many related methods have been proposed in this field. However, most of them are based on single-frame prediction, which causes unstable results between frames. Some semantic multi-frame segmentation methods produce error accumulation and are not fast enough. Therefore, we propose a deep learning algorithm that takes into account the continuity information of adjacent image frames, including image sequence processing and an end-to-end trainable multi-input single-output network to jointly process the segmentation of lanes and road markers. In order to emphasize the location of the target with high probability in the adjacent frames and to refine the segmentation result of the current frame, we explicitly consider the time consistency between frames, expand the segmentation region of the previous frame, and use the optical flow of the adjacent frames to reverse the past prediction, then use it as an additional input of the network in training and reasoning, thereby improving the network’s attention to the target area of the past frame. We segmented lanes and road markers on the Baidu Apolloscape lanemark segmentation dataset and CULane dataset, and present benchmarks for different networks. The experimental results show that this method accelerates the segmentation speed of video lanes and road markers by 2.5 times, increases accuracy by 1.4%, and reduces temporal consistency by only 2.2% at most.

## 1. Introduction

Unmanned driving technology or auxiliary driving technology has broad development prospects. The sensing module and the control unit constitute an unmanned driving system [1]. The precondition of stable operation of unmanned driving systems is the understanding and recognition of high performance environments, which depends on the sensing module composed of multiple sensors [2]. The lanes and road markers on the highway do not have special three-dimensional shapes, and collecting the geometric contour of the surrounding environment only by relying on radar is not enough [3]. Therefore, in addition to radar, visual sensors and computer vision technology are important links in the whole sensing module [4]. Moreover, only algorithms that can maintain robustness and achieve high-quality real-time performance under different circumstances and environments are suitable for unmanned driving systems [5].

In recent years, increasing numbers of studies have been conducted on semantic image segmentation based on on-board RGB cameras in academia and industry. With the powerful abstract ability of deep learning to learn image features, segmentation of lanes and road markers has achieved good results, and has gradually become a mainstream technology in today’s research [6]. However, most of the research on the segmentation of lanes and road markers is limited to the segmentation of a single image, which produces unstable segmentation results in the segmentation of continuous video sequences. Although previous methods presented state-of-the-art results in a single frame of an image, they produced crude extrapolations and had efficiency and robustness issues [7]. In particular, the human eye is able to identify lanes and road markers that are hard to see, allowing drivers to drive safely [8]. The reason why the driver can drive in this situation is that the human eye not only analyzes the pictures at every moment they see, but also makes full use of the effective spatio-temporal information between the sequence of pictures in successive moments [9]. In other words, the current judgment is highly related to past information [10]. Some common semantic video segmentation methods consider the correlation of video sequences, for example, the method based on key frames: in every few frames in sequence, it selects a frame as a key frame. This method can make full use of repeated sequences of frame information, but increasing distance from the current frame and the key frames may lead to the accumulated error of semantic segmentation becoming more serious.

In order to consider how to make full use of sequence information, we thought that the position of the target in adjacent frames in a video sequence does not mutate. Inspired by this, we intended to contribute to autonomous driving research by:

- Providing two forms of network prior information. One is image preprocessing using dilation and erosion. Before the image is input into the network, we use the pre-frame segmentation as a mask to crop the image, and the cropped image is used as one of the inputs of the network. The purpose is to identify the location of the target in the picture with high probability. In the other one, the optical flow between adjacent frames is calculated. Considering the validity of adjacent information, the segmentation result of the previous frame warps to the current frame position as another input of the network.
- Designing an end-to-end trainable multi-input single-output network that uses multiple prior information to jointly process the segmentation of lanes and road markers. Additionally, no post-processing is introduced to avoid the extra computation cost caused by post-processing, and real-time segmentation without frame delay can be achieved.
- Evaluating our network in detail in benchmarks on the Apolloscape dataset and the CUlane dataset, and the experimental results show that our algorithm can output smoother results in video sequences, and has better robustness than other algorithms, as well as better real-time performance. In particular, the lane and road marker targets that are difficult to observe in the images can also be segmented by our algorithm.

The remainder of this paper is structured as follows: Section 2 describes the research on processing lane and road marker problems in recent years as well as the research on general video semantic segmentation. The third section describes our proposed model in detail. Section 4 presents the experimental content and results. Finally, we summarize the work of this paper in the final section.

## 2. Related Work

In this section, we introduce some of the previous studies on lanes and road markers, as well as general video semantic segmentation.

In recent years, the detection and segmentation of lanes or road markers based on deep learning technology have considerably developed. Huval et al. [11] and Kim et al. [12] proposed using a convolutional neural network (CNN) to deal with lane detection. Li et al. [13] used CNN to extract the geometric feature information of lanes in pictures, and then detected lane boundaries through a recurrent neural network (RNN). Neven et al. [14] proposed Lanenet, which reduced the lane detection problem to an instance segmentation problem, in which each lane formed its own instance and could be trained end-to-end. Qin et al. [15] proposed a new method for lane detection based on CNN. Different from per-pixel image segmentation, this method directly predicts the position of each lane in the input image with a small amount of calculation. Pan et al. [16] proposed a new lane detection network structure. Different from the convolution operation commonly used, the network convolved the feature map in four directions in order to extract features. Jie et al. [17] combined lane detection and drivable area detection in one task, and used the clear geometric relationship between the two tasks to exchange effective features between the two subnetworks of the multi-task network. Lee et al. [18] established a new dataset containing harsh environment image samples and proposed an end-to-end trainable multi-task network to simultaneously solve the detection and recognition of lanes and ground indicator arrows. Liao et al. [19] proposed a context aggregation module (CAM) and a spatial detail module (SDM) to adaptively encode multi-scale context information and channel context information in a larger receptive field, and improve the transmission efficiency of low-frequency information from a low to a high level. Sediqi et al. [20] proposed dense upsampling convolution (DUC) and dense local context (DLC) to generate denser high-resolution feature representations and effectively extract the context information of objects in the scene.

Although the above deep-learning-based methods can achieve good performance in the task of segmenting lanes and road markers in single frame image, they do not consider the effective information between frames of video sequences, and produce unstable segmentation results in the segmentation of continuous video sequences.

Semantic segmentation based on video sequences requires pixel-level classification of every frame image in a video; that is, each pixel in each image is classified into several semantic categories. Fayyaz et al. [21] proposed an end-to-end architecture combining temporal and spatial characteristics, and connected multiple fully convolutional networks (FCNs) into a spatio-temporal convolutional network using long short-term memory (LSTM). Nilsson et al. [22] designed a spatio-temporal Transformer Johnson unit to integrate the information contained in regions with large differences between frames (drift deformation). Jin et al. [23] predicted the current image and segmentation through the previous four frames, and then predicted the segmentation result of the current frame by combining the features of the previous four frames and the current frame with the timing information. Gadde et al. [24] proposed Netwarp, which uses the optical flow between adjacent frames to warp the feature image in the previous CNN frame and add it to the CNN of the current frame. This method is applicable to many CNN-based network architectures. Li et al. [25] proposed an efficient and low-latency semantic video segmentation algorithm that can adaptively propagate interframe features and schedule key frame positions. Liu et al. [26] proposed a timing distillation method based on time consistency and a loss function considering inter-frame consistency. Without the introduction of post-processing methods, this method can be used for many network models by balancing accuracy, timing consistency, and efficiency without increasing the original model’s inference time. Lu et al. [27] used optical flow estimation to optimize the lane segmentation of consecutive frames, and proposed an adaptive scheduling network (ASN), which can be used to determine the working module (segmentation network or optical flow tracking module) based on the downsampling feature map of the optical flow estimation network.

In order to bridge the gap in the field of view and structure distribution between imaging domains, Yang et al. [28] introduced an efficient concurrent attention network called efficient concurrent attention networks (ECANets), which include a horizontal segment attention (HSA) module and a pyramidal space attention (PSA) module, directly capturing the long-range dependencies inherent in omnidirectional imaging and reducing the amount of calculation required by the attention mechanism.

In order to overcome the limitations of the CNN system on the receptive field, Zheng et al. [29] proposed using a transformer to extract global features and added a decoder for semantic segmentation. The CNN structure is more suitable for low-level features, and the transformer is more suitable for high-level semantics. The segmentation task not only requires deep semantic information, but also low-level texture boundary information to achieve accurate segmentation of object boundaries. Referring to the research of Dosovitskiy et al. [30], the transformer needs large-scale data for training so that better deep semantic information of the image can be obtained, and the transformer needs to perform multiple self-attention calculations and requires a large amount of computing resources. Liu et al. [31] proposed CondLaneNet, using ResNet [32] as the backbone network, adding the standard FPN [33] as the net, and adding a transformer to the minimum feature map output by ResNet to capture the information associated with lane lines. In the proposal head part, CondLaneNet divides the image into several grids and outputs two feature maps: one is a heatmap, which indicates whether there are lane line instances in each grid; and the other is a parameter map, which outputs a set of parameters corresponding to the lane line instances. Then, on the feature map of the shared branch, the previously calculated convolution parameters are used to calculate location maps and offset maps. Location maps are single-channel feature maps. For each row of the feature map, SoftMax is first performed, and then the output of SoftMax and the column coordinates are weighted and summed to obtain the lane line position loc of the row. At the same time, for each row of the feature map, a fully connected layer is used to predict whether the lane line crosses the row. Since the calculation of loc is not accurate enough, offset maps are used to predict an offset value in the horizontal direction.

In this paper, we propose an algorithm that can fully use the sequence interframe information and can achieve higher accuracy in the segmentation of lanes and road markers of video sequences, suppress the instability in the segmentation of continuous video sequences, reduce the waste of interframe information, and achieve a trade-off between accuracy and efficiency.

## 3. Model

### 3.1. Architecture Overview

**Figure 1:**
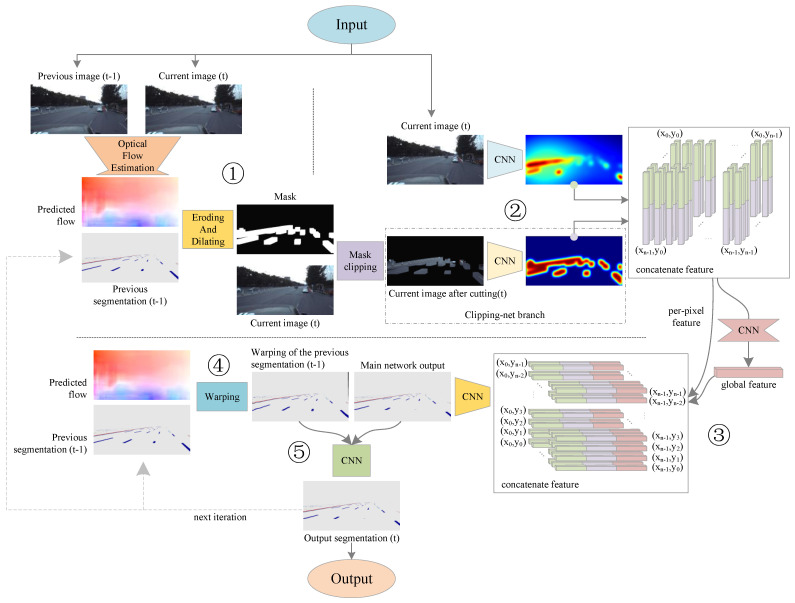
Overview of our model. A mask is generated by the optical flow of adjacent frames to crop the current frame image, and the cropped image and the complete image are used as the input of the main network, which is responsible for the output of the preliminary prediction of the current frame. Then, the optical flow of adjacent frames is used to warp the segmentation of the previous frame to the current frame position, and the distorted segmentation and the output of the main network are used as the input of the optimization network, which is responsible for revising the preliminary prediction of the main network and output the final segmentation of lanes and road markers.

Figure 1 shows the overall architecture of our algorithm. Our algorithm mainly consists of five logical steps.

The first step is to obtain the optical flow information. In this stage, the color images of the current frame and the previous frame are used as the input, and an optical flow estimation network is used to calculate the optical flow between the two frames.

In the second step, the segmentation result of the previous frame and the color image of the current frame are input. The segmentation result of the previous frame after the erosion and dilation operation is used as a mask to crop the color image of the current frame, and the cropped image and the complete image are input to the third step.

The third step involves inputting the cropped image and the complete image, and extracting two different color features. Preliminary predictions of lanes and road markers in the current frame are output through special feature fusion and feature extraction methods. In this paper, we call the network in this step the main network.

The fourth step is warping the segmentation result output from the previous frame to the current frame position according to the optical flow information between adjacent frames.

The fifth step is connecting the preliminary prediction result of the current frame output in the third step with the warped segmentation result of the previous frame output in the fourth step, and then correcting and outputting the segmentation result of the final lanes and road markers of the current frame through CNN. In this paper, we call the network in this step the optimized network.

**Figure 2:**
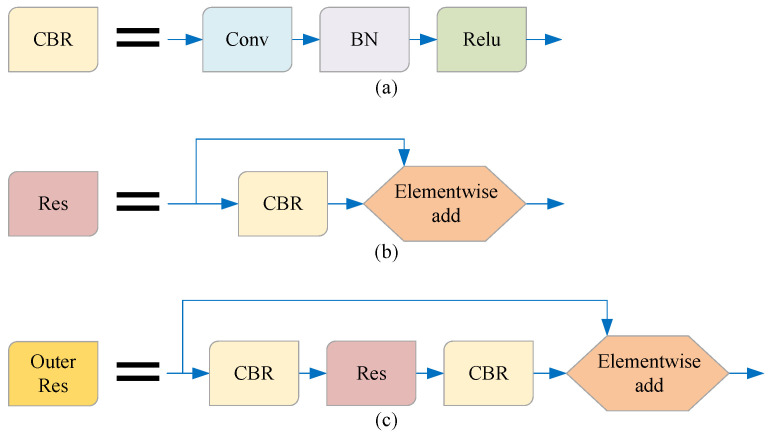
The names of some basic network combinations are simplified in this paper. (**a**) The combination of the convolution layer, BN layer, and ReLU activation function is referred to as the CBR unit; (**b**) the combination of the CBR unit and residual structure is referred to as the RES unit; (**c**) the combination of CBR-Res-CBR and residual structure is referred to as the Outer Res unit.

As shown in Figure 2, in order to facilitate the introduction of our model, we simplify the terms of some basic network combinations in this paper. The method in this paper is described in detail below.

### 3.2. Optical Flow Estimation

The key to this step is estimating the optical flow of the current frame relative to the previous frame. We take two images of the same size as the input, and estimate the moving distance and direction of the optical flow of each pixel in the two-dimensional plane of the image. The main aim in this study was to design a segmentation algorithm for lanes and road markers. Therefore, we used the optical flow estimation network PWC-NET proposed by [34] to directly obtain optical flow information.

Optical flow estimation is performed offline; PWC-NET is trained separately, and participates in inference after training.

### 3.3. Mask and Crop

In a video sequence, the position of the target in the adjacent frame does not change suddenly; that is, the translation and rotation of the target existing in the previous frame in the current frame image are within a certain range. Therefore, our method involves using the output of the previous frame to emphasize the area with a high probability of occurrence of the target in the current frame.

In order to reduce the influence of noise, we first corrode the segmentation result of the previous frame. The function of the erosion operation is to reduce the segmentation noise, so the size of the erosion core should be set very small; otherwise, the small correct segmentation will be erased by mistake. We set the size of the erosion core to 1 × 1.

**Figure 3:**
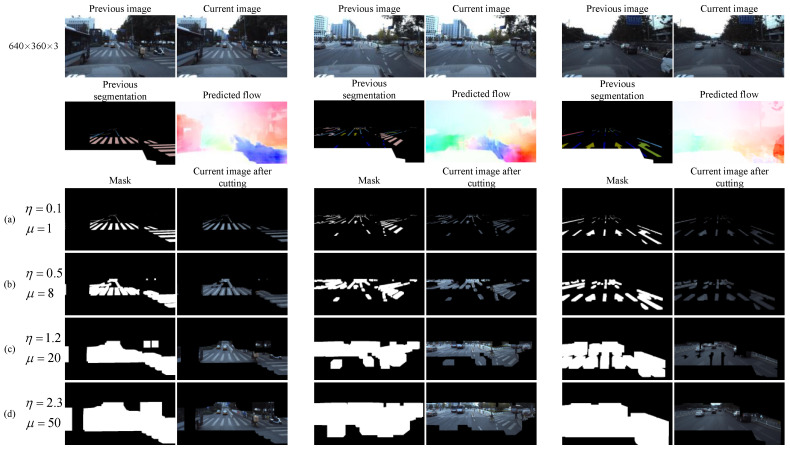
The effect of cropping images with four different dilation kernel sizes. (**a**) The global size of the dilation kernel is approximately 1; that is, the cropped area is approximately equal to the segmentation result of the previous frame. It is obvious that the cropped image cannot cover the target position of the current frame; (**b**) the size of the dilation kernel is expanded, and the cropped image can cover more of the current frame target region, but it has not reached complete coverage. (**c**) Further expanding the size of the dilation kernel, the cropped image can completely cover the target region of the current frame. (**d**) Continue to expand the size of the dilation kernel. Obviously, the cropped image can completely cover the target area of the current frame, but too much redundant background is retained.

Then, we expand the segmentation area through the dilation operation to cover the possible position of the target in the current frame image and provide a region proposal. We use the rectangular dilation kernel; the size of the dilation core should be determined according to the motion intensity of the adjacent frame scene. There are many factors that affect the motion intensity of adjacent frames, such as the video frame rate, the linear velocity and angular velocity of the target movement, and the linear velocity and angular velocity of the camera movement. In order to avoid the coupling of these unrelated factors, we calculate the dense optical flow between adjacent frames in the first step, and determine the size of the dilation core through the optical flow. Specifically, we first calculate the optical flow of the entire image, and then evenly divide the image into S × T grids, where each grid uses a different dilation core size. In order to cover the positions of the target in the current frame image as much as possible, the size of the dilation core in a grid is determined by the maximum optical flow distance in the grid, and the calculation method is as follows:

(1) $$d=\sqrt{\beta_{1}^{2}+\beta_{2}^{2}}$$

(2) $$D=\eta \cdot \max(d_{1},d_{2},\cdots ,d_{n})+\mu$$

where *D* is the size of the dilation kernel in the grid; *d* is the Euclidean optical flow distance of the pixel points in the grid, $\beta_{1}$ and $\beta_{2}$ are the transverse optical flow distance and longitudinal optical flow distance of the pixel points, respectively; *n* is the number of pixel points in the grid; and η and μ are parameters that are used to fine-tune the size of the dilation kernel. Figure 3 shows a comparison of the dilation effect and image cropping effect generated by four typical values of η and μ in the 640 × 360 image in three scenes.

### 3.4. The Main Network

#### 3.4.1. Extract the Characteristics of Different Input Sources

**Figure 4:**
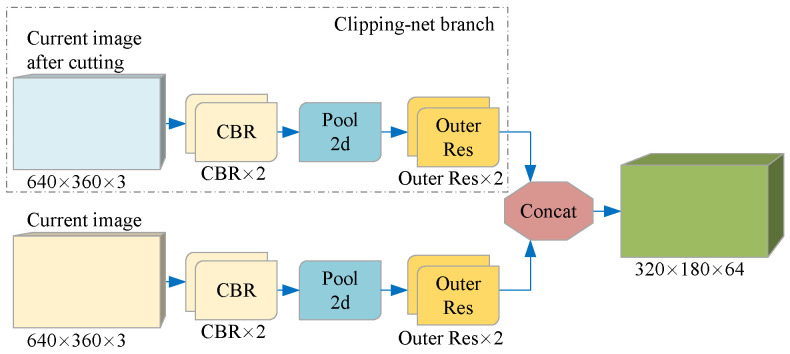
Per-pixel color features of two different data are extracted on two branches. Each branch is a CNN-based structure, which uses a continuous 3 × 3 convolution layer and residual network structure to map a color image of size W × H × 3 to a feature space of size W/2 × H/2 × C. Each element in the feature space is a C-dimensional vector representing the semantic information of the input image on the corresponding coordinate.

The cropped image tends to detect the position of the existing target in the current frame, while the complete image is used to supplement the position of the new target. Although the cropped image and the full image of the current frame are in the same RGB format, the information they contain has different degrees of importance. Therefore, instead of directly connecting the two images, we introduce a branch we call Clipping-net to process the cropped images separately. As shown in Figure 4, CNN extracts the color features of the cropped image and the complete image on two branches separately to retain the inherent information weight of the two different data and finally connect them together.

#### 3.4.2. Fusion Features and Output Preliminary Predictions

The color features were extracted from the cropped image and the complete image above, and now these features need to be merged. A commonly used method is to directly generate pixel-by-pixel predictions from the trimmed image features and complete image features. However, sometimes, due to cropping errors, the previous cropping may be incomplete or contain too many background pixel features. Therefore, directly fusing the two features will reduce the prediction performance.

**Figure 5:**
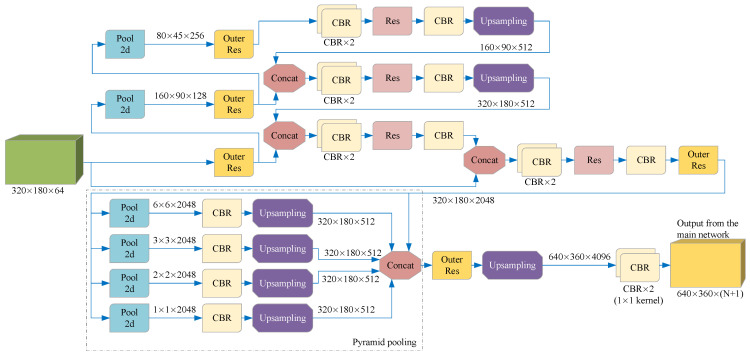
A network for feature fusion and output preliminary prediction. We trained this network to predict the preliminary lane and road marker segmentation according to the cropped image and the complete image, output N + 1 two-dimensional matrices with the same length and width as the color image, and then obtained the preliminary prediction according to SoftMax. Each element of the same pixel coordinate in the N + 1 matrices represents the probability that each pixel in the current frame image is predicted to be N categories or backgrounds.

As shown in Figure 5, we use a special feature fusion method suitable for the segmentation of lanes and road markers when the cropped image contains too much background or the cropped image is accidentally incomplete. The key idea is to carry out global feature fusion before generating per-pixel prediction, so that the prediction can be provided according to the semantic information of the whole feature space; that is, the complete information of the whole color image can be considered implicitly, and the impact of image cropping errors can be reduced. Specifically, we first connect the feature maps of the two input images on the channel dimension, and then input them into a CNN containing a pyramid structure, using the code-decoding structure to generate a global feature map with a constant size. Then, the feature maps directly connected with the two features are connected to obtain the complete feature maps. We use the complete feature maps to enrich the features of each pixel to provide global context information. Then, the resulting complete feature map is input into the Pyramid pooling network instead of directly upsampling in order to better use the global feature information and avoid mismatching, category confusion, and neglecting of small categories. The output of the Pyramid pooling network is then entered into a decoder consisting of a 3 × 3 convolutional layer, a 1 × 1 convolutional layer, an upsampling, and a residual structure to output the preliminary lanes and road markers prediction results. In this paper, this part and the above part of the network are collectively referred to as the main network.

### 3.5. Warp the Segmentation Result of the Previous Frame

We use the optical flow information estimated in the first step to warp the segmentation result output from the previous frame to the current frame position. The transformation of any pixel from the old two-dimensional coordinates to the new two-dimensional coordinates is:

(3) $$\left\{\begin{array}{c} x=\delta_{11}v+\delta_{21}w+\varsigma_{1}\\ y=\delta_{12}v+\delta_{22}w+\varsigma_{2} \end{array}\right.$$

where *x* and *y* are the new abscissa and new ordinate after pixel transformation, respectively; and *v* and *w* are the old abscissa and old ordinate before pixel transformation, respectively. The corresponding homogeneous coordinate matrix is expressed as:

(4) $$\left[x,y,1\right]=\left[v,w,1\right]\cdot T=\left[v,w,1\right]\cdot \left[\begin{array}{ccc} \delta_{11} & \delta_{12} & 0\\ \delta_{21} & \delta_{22} & 0\\ \varsigma_{1} & \varsigma_{2} & 1 \end{array}\right]$$

where the pixel space coordinate scaling rotation transformation matrix and translation transformation matrix are:

(5) $$\lambda =\left[\begin{array}{cc} \delta_{11} & \delta_{12}\\ \delta_{21} & \delta_{22} \end{array}\right]=\left[\begin{array}{cc} 1 & 0\\ 0 & 1 \end{array}\right],B=\left[\varsigma_{1},\varsigma_{2}\right]$$

Our warping processing needs to preserve the original size and scale of the target, so the scaling transformation matrix λ is the identity matrix *E*. The values of $\varsigma_{1}$ and $\varsigma_{2}$ in matrix *B* are the two elements in the last dimension of the four-dimensional matrix generated by optical flow estimation, respectively representing the transverse and longitudinal movement distances of post-warping coordinates relative to pre-warping coordinates.

### 3.6. Optimized Network

**Figure 6:**
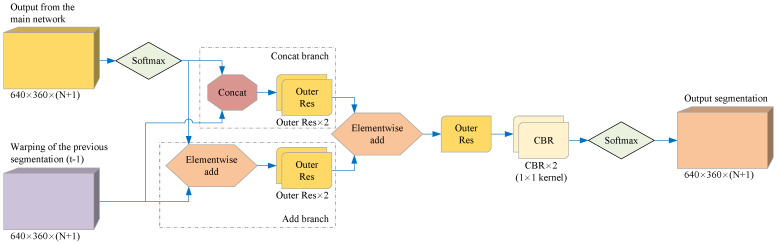
The structure of the optimized network. Our optimized network uses two feature map fusion methods to improve the main network segmentation results, so it contains two branches: a Concat branch and an Add branch. Finally, the optimized network outputs the segmentation results through SoftMax.

In order to fully use the continuous information of the video sequence, we combine the preliminary detection result of the current frame output by the main network with the segmentation result of the previous frame after optical flow warping, so that the model can correct the segmentation of the current frame. The key is to train the network to improve the initial segmentation results instead of making new predictions. For this, we must use the segmentation of the previous frame as part of the input for this stage. Our main idea is using CNN to learn the local information of adjacent frames and output more refined detection results. For the convenience of description, we call the network at this stage the optimized network. Figure 6 shows the structure of the optimized network.

The optimized network can be trained together with the main network, but in the initial stage of training, the output effect of the main network is poor; that is, it contains too much noise, which affects the learning efficiency of the optimized network and leads to the failure of the optimized network to learn enough meaningful information. Therefore, in the experiment, it was often necessary to let the main network train and converge first, and then we started the joint training.

### 3.7. Loss Function

After defining the entire network structure, we focused on the training objectives. As mentioned above, our method first needs to train the main network, and then the optimized network is added to the training. We used Focal Loss in both training stages.

We define the cross-entropy of all the pixel coordinates of the lanes and road markers in the label and the corresponding pixel coordinates of the lanes and road markers predicted by the network as the loss. Different from general image semantic segmentation tasks, in tasks of segmenting lanes and road markers, most of the area in the image is the background: the number of background pixels is about 100 times the sum of the number of pixels in other categories. Therefore, the category probability of the network output is easily dominated by a large area of background. In order to weaken the dominant position of the background category in the image on the training loss, we expanded it into multi-classification Focal Loss based on the binary loss function Focal Loss proposed in [35]. Specifically, the loss of network is defined as:

(6) $$L=\frac{1}{H\cdot W}\sum_{x=0}^{H}\sum_{y=0}^{W}\left[-\alpha_{(x,y)GT}\cdot {\left(1-P_{(x,y)GT}\right)}^{\gamma }\cdot \log P_{(x,y)GT}\right]$$

where (x,y) represents the pixel coordinates; *H* and *W* represent the height and width of the network output size, respectively. Since the network output size is the same as the network input size, they also represent the height and width of the network input size. *p* represents the probability of (x,y) pixel being predicted as the true category, γ is used to weaken the dominant effect of high-probability categories on the overall loss, α represents the true category weight of (x,y) pixel, and all category weights are dimension vectors:

(7) $$A=[\alpha_{1},\alpha_{2},\alpha_{3},\cdots ,\alpha_{n-1},\alpha_{n}]$$

where *n* is the number of categories, and the value of α is determined according to the proportion of the number of samples of this category:

(8) $$\alpha_{class}={\left(1-P_{class}\right)}^{\delta }$$

where $P_{class}$ represents the proportion of the number of samples in this category, and δ is used to weaken the dominant effect of the category with a high proportion on the overall loss, that is, to reduce the loss of samples with high proportion.

In the joint training stage of the main network and the optimized network, we calculated the Focal Loss of the main network and the optimized network, separately. The complete loss function of joint training is the average of the two losses:

(9) $$L_{ours}=\frac{L_{m}+L_{o}}{2}$$

where $L_{ours}$ refers to the complete loss of joint training, $L_{m}$ refers to the network loss, and $L_{o}$ is the optimized network loss.

## 4. Experiment and Analysis

Our experimental platform was configured as follows: Intel i9-9900K 5 GHz × 8 CPU, 32 GB RAM, NVIDIA Tesla V100 32 GB GPU (NVIDIA, Santa Clara, CA, USA).

### 4.1. Dataset and Evaluation Metric

**Figure 7:**
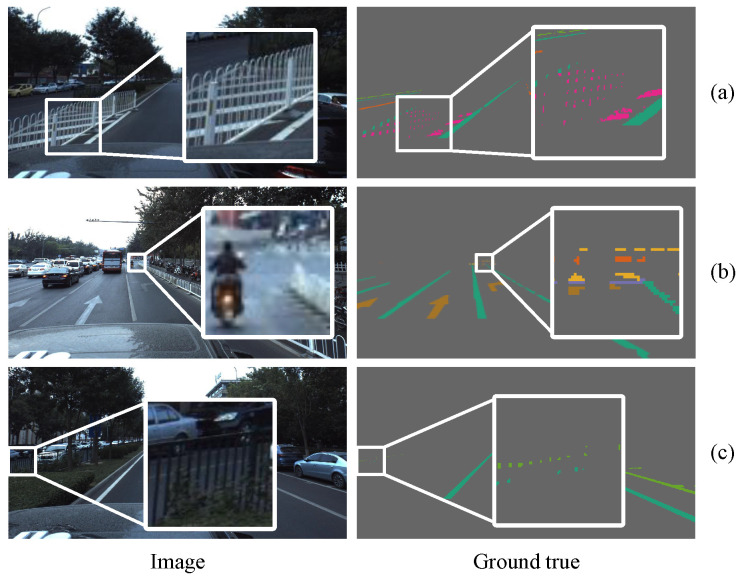
The annotations of the Apolloscape lanemark segmentation dataset are very accurate. (**a**) Safety zone warning lines in fence gaps; (**b**) tiny targets in the distance, including turning arrows, zebra crossings, stop lines, and single solid lines; (**c**) single solid lines of opposite lanes and border lines of parking areas obscured by railings.

**Figure 8:**
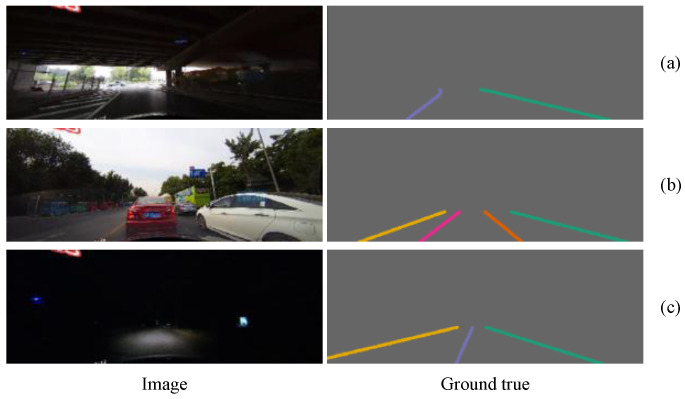
The CULane dataset contains a variety of challenging scenarios: (**a**) the ratio of light to dark in the image under the overpass is very large; (**b**) the traffic on the road is crowded, and the blocked lanes are also marked according to the context; (**c**) the night scene is extremely dark, and the visibility of the lanes is poor.

We used Baidu’s open-source Apolloscape lanemark segmentation dataset [36] and the CULane dataset [16] to test our model. The Apolloscape lanemark segmentation dataset wasa collected from many cities in China, such as Beijing, Shanghai, and Shenzhen. The Apolloscape lanemark segmentation dataset contains tens of thousands of labeled images with a resolution of 3384 × 2710, covering solid lines, broken lines, turning arrows, zebra crossings, parking area boundaries, and covering a variety of different traffic scenes. In particular, the labels of the Apolloscape lanemark segmentation datasets were generated on a three-dimensional point cloud and then projected onto a two-dimensional image plane, so it is very precise. As shown in Figure 7, even the seemingly small targets in two-dimensional images are labeled. The dataset also includes complex conditions such as different weather conditions, specular reflections of objects, over- or under-exposure, congestion and obstructing of vehicles or pedestrians, and damaged targets. CULane is a large-scale challenging dataset for academic research on lane detection. It was collected by cameras installed on six different vehicles driven by different drivers in Beijing, who collected more than 55 h of video and extracted 133,235 frames. For each frame, CULane manually annotates the lane lines using cubic splines. As shown in Figure 8, when the lane markings are obscured or invisible by the vehicle, CULane still marks the lanes according to the context.

In training, we doubled the amount of data by flipping the image horizontally to avoid model prediction bias caused by the left or right perspective. In other words, the Apolloscape lanemark segmentation dataset and the CULane dataset were collected from the road driving on the right, and we can simulate road driving on the left by flipping the dataset horizontally. In particular, for classes whose meanings change after horizontal flipping, we also amended their class labels at the same time.

We used the method proposed in [26] to evaluate temporal consistency (TC). The segmentation of the previous frame is warped to the current frame position by optical flow, and the intersection over union (IoU) between the two segmentations is calculated as the evaluation of TC. The calculation method is as follows:

(10) $$TC\left(P_{t-1},P_{t}\right)=\frac{\hat{P_{t-1}}\cap P_{t}}{\hat{P_{t-1}}\cup P_{t}}$$

where $P_{t}$ is the segmentation result of the current frame, and $\hat{P_{t-1}}$ is the result after the segmentation of the t−1 frame is warped to the position of the current frame. Then, the average TC of each sequence in the test set was calculated to evaluate time consistency, as follows:

(11) $$TC=\frac{1}{M}\sum_{i=1}^{M}\frac{\hat{\rho_{i}}\cap \rho_{i}}{\hat{\rho_{i}}\cup \rho_{i}}$$

where $\hat{\rho }=\left\{\hat{P_{1}},\hat{P_{2}},\cdots ,\hat{P_{T-1}}\right\}$ and $\rho =\{P_{2},P_{3},\cdots ,P_{T}\}$, *T* is the number of images in the video sequence, and *M* is the number of video sequences in the test set.

For the CULane dataset, we used the evaluation method mentioned in SCNN [16], which calculates the F1 measure as the evaluation criterion. By calculating the IoU between the ground truth and prediction, a threshold is set to determine whether the prediction is true positive (TP), false positive (FP), or false negative (FN). The IoU between ground truth and prediction was defined as the IoU between two fixed-width lanes. Specifically, the calculation method of the F1 measure is as follows:

(12) $$Precision=\frac{TP}{TP+FP}$$

(13) $$Recall=\frac{TP}{TP+FN}$$

(14) $$F1measure=\frac{2\cdot Precision\cdot Recall}{Precision+Recall}$$

### 4.2. Training

In the experiment, we found that the effect of directly training the whole model was often poor because the output of the main network contained too much noise in the initial stage of training, which disrupted the learning ability of the optimized network, resulting in the inability of the optimized network to learn enough meaningful information. Therefore, our training work was divided into two stages. First, we only trained the main network to initialize the parameters, and then, after the convergence of the main network, we added the optimized network into the joint training. For both stages, the model is trained by the Adam optimizer [37] controlled by the β parameter. We set $\beta_{1}=0.9$, $\beta_{2}=0.99$, and the basic learning rate as $2\times 10^{-3}$.

**Figure 9:**
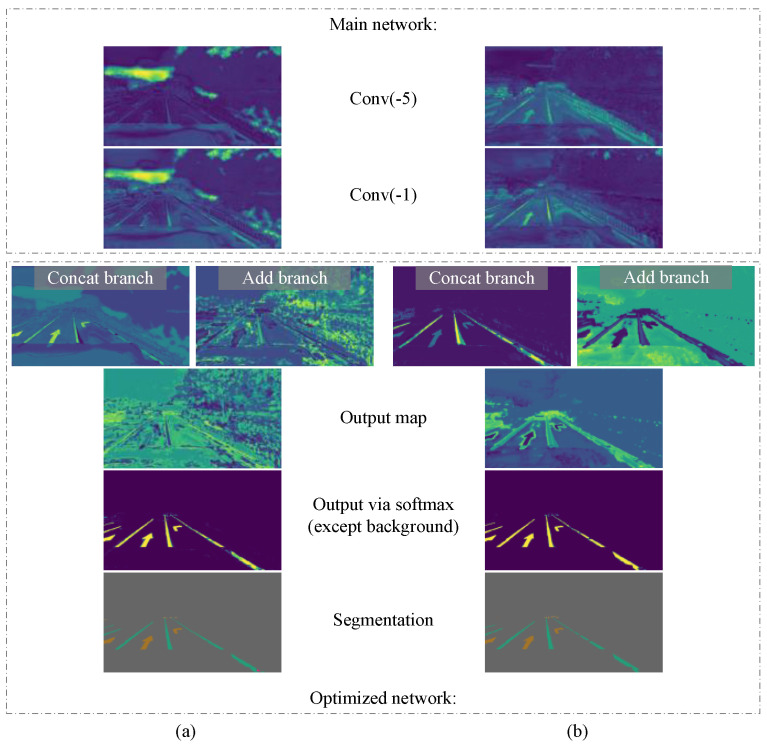
Output and intermediate feature maps of our model under two training strategies. (**a**) The model with direct joint training from start to finish. The main network cannot focus on the lane and road marker targets, and the optimized network produces a large amount of noise. (**b**) The model in which the main network is trained first and then they are trained together. The main network can extract the target features correctly, and the optimized network produces only a small amount of noise, and the model output is better.

In order to observe whether this training strategy was conducive to improving the learning effect of our model, we compared two training methods, and the experimental results are shown in Table 1. We also output the middle layer feature maps of the model separately, as shown in Figure 9. Obviously, more neurons respond correctly and produce less noise, especially near lanes and road markers, indicating that the network using this training strategy can extract target features faster.

### 4.3. Ablation Experiments

First, we separately tested the improvement of the Clipping-net branch in the network for lane and road marker segmentation, and we tried different dilation kernel sizes. In Table 2, we show the changes in the mean intersection over union (mIoU) and TC caused by the size adjustment of the dilation kernel of mask cropping. It can be seen that in the forward reasoning process of the model, a small dilation kernel leads to poor effect, and mIoU and TC increase with the expansion in the size of the dilation kernel. However, when η and μ exceeded 1.2 and 25, respectively, the model could not continue to obtain more gains, and even showed a decrease in mIoU and TC.

**Figure 10:**
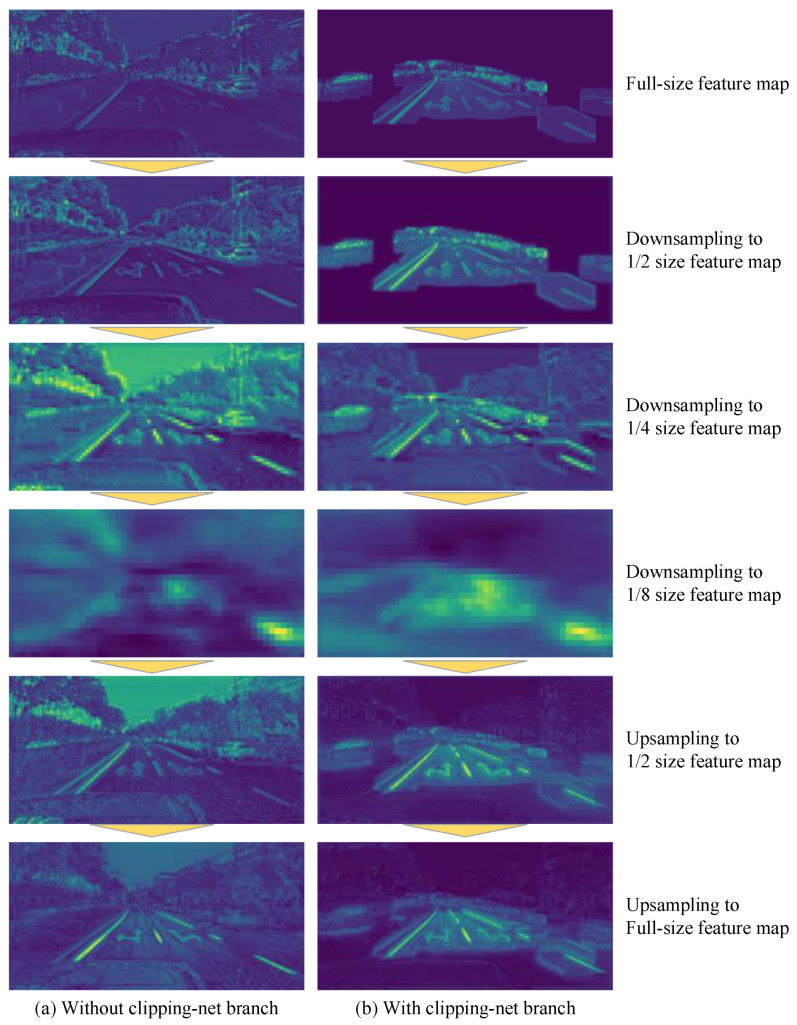
The feature maps of different sizes output by convolutional layers of different depths in the network. (**a**) Our network without Dlipping-net branch: the input of the network is only the complete image of the current frame. Some convolution kernels react to many non-target features, resulting in features near lanes and road markers being difficult to be extracted correctly by the network. (**b**) Our network with Clipping-net branch: the inputs of the network not only include the complete image of the current frame, but also the cropped image of the current frame. Obviously, the Clipping-net branch can inhibit the activation of the convolutional kernels in the background. Convolutional kernels almost never react to non-target features, and most of the activated neurons are concentrated near the target. Compared with (**a**,**b**) can extract the features near lanes and road markers earlier.

In Table 3, we show the classes’ mIoU of the networks with Clipping-net branch and the networks without Clipping-net branch. Our network achieved the best mIoU performance when we set η=1.2 and μ=25 so that the dilation kernel size was 1.2 times the optical flow distance plus half of the average width of the lanes and road markers (about 25 pixels). Notably, the networks without Clipping-net branch and with η=2.8 and μ=50 as the Clipping-net branch network have an almost mIoU average of the input, but the precision of each category is different, and the linear target segmentation accuracy increases, apparently because the Clipping-net branch provides more information, i.e., the location of the target may exist in the current frame. We output feature maps of different sizes output by the convolution layers of different depths in the main network with and without Clipping-net branch. We averaged the feature values of each channel in the feature maps. These feature maps are shown in Figure 10. It can be easily observed that when we added Clipping-net branch into the main network, the features near the lanes and road markers in the feature map were more obvious; that is, the main network could extract the correct features in the shallower convolution layer. Therefore, in the other experiments that followed, we added the Clipping-net branch to the main network and used η=1.2 and μ=25.

### 4.4. Evaluation

#### 4.4.1. Apolloscape Lanemark Segmentation Dataset

We also tested other algorithms for comparison, including the single-frame image algorithm PSPNet [38], VPGNet [18], DeepLabv3+ [39], Dilation-CNN [40], HRNet [41] and the semantic video segmentation algorithm [26], GRFP [22], and NetWarp [24]. Table 4 shows the comparative results. All the listed algorithms used NVIDIA Tesla V100 to retrain and predict on the Apolloscape lanemark segmentation dataset. We used the best single-frame method DeepLabv3+ [39] and the best multi-frame method PSPNet + Eff. [26] as the baseline to compare the performance on the test set. Compared with the single-frame image segmentation method, the TC of all the semantic video segmentation methods increased. Compared with the high-precision, single-frame image semantic segmentation algorithm DeepLabv3+, our algorithm produced a 1.5% improvement in mIoU classes, 1.1% improvement in mIoU categories, and 6.5% improvement in TC, and the speed was increased three-fold. The TC of our algorithm was 2.2% lower than the best semantic video segmentation algorithm PSPNet + Eff. [26], but produced a 1.4% improvement in mIoU classes and a 1.5% improvement in mIoU categories, and the speed was increased 2.5-fold.

**Figure 11:**
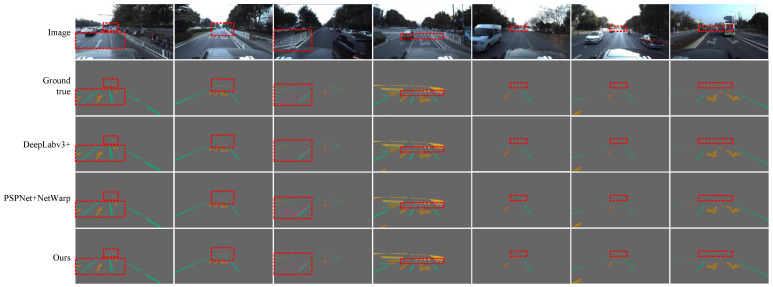
Some examples of the segmentation results of lanes and road markers on the Apolloscape lanemark segmentation dataset by our model are presented. Our model can obtain more information from adjacent frames and segmentation better by using clipping-net branch and optical flow optimized network. We indicate the obvious segmentation differences between the different methods with a red dotted box.

We show some examples from the test set in Figure 11 to illustrate the benefits of our approach. Notably, our method can suppress the instability caused by the segmentation of continuous video sequences and more accurately segment the turn arrows, broken lines, zebra crossings, and the opposite lane blocked by railings. It can be found that the positions of the above targets are obviously changed by inter-frame motion. When we use prior information to emphasize the possible positions of the targets and combine the consistent information of adjacent frames together, the segmentation of the current frame becomes easier and more accurate.

We also noticed that, compared with the above targets, the positions of single solid lines and double solid lines were insensitive to inter-frame motion, so their segmentation accuracy was already very high in the single-frame lane and road marker segmentation methods, so cannot be easily improved by the current proposed semantic video segmentation methods, and our method is no exception.

#### 4.4.2. CULane Dataset

**Figure 12:**
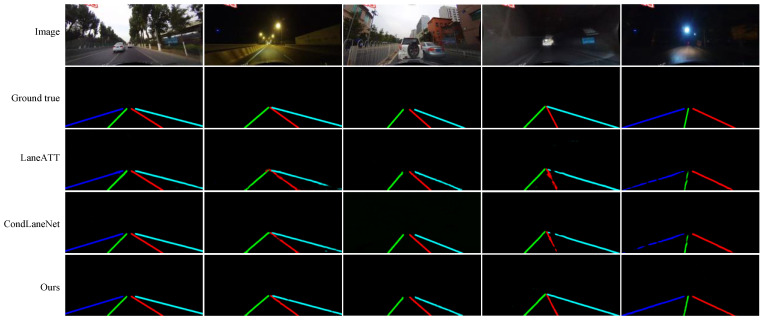
Some examples of the qualitative comparison results of our work on the CULane dataset are shown. In some difficult scenarios, because our method explicitly considers the segmentation in the previous moment, it can sometimes produce better results.

We provide additional experimental comparisons on CULane. In Table 5, compared with the baseline method, our lane line segmentation accuracy and time consistency are improved, and a better trade-off is achieved between accuracy and inference speed. Obviously, our method produced improved performance in dazzle light, arrow, and night scenes. This is because the lanes in these scenes become difficult to detect. If only relying on the image of the current frame for inference, segmentation becomes very difficult. Our method considers the past segmentation and the optical flow between image frames and uses them to optimize the segmentation accuracy of the current frame, thus making lane segmentation easier. In addition, TC considerably improves. The advantage is that the segmentation result of the sequence is more stable, and the segmentation jitter of different frames is reduced. Some qualitative comparisons are shown in Figure 12.

However, we also found some limitations of our method: for normal or other scenes, our method cannot improve accuracy. This is because, in these scenes, the lanes are clearly visible in most cases; in other words, the inter-frame movement has little effect on the target position. Our method is obviously aimed at scenes that are occluded or difficult to see in lanes and the stability of sequence segmentation, so our method does not improve the segmentation accuracy in normal or other scenes.

## 5. Conclusions

One of the important technologies in the field of autonomous driving is the segmentation of lanes and road markers. Previous researchers have proposed many methods for this task. Most of the lane and road marker segmentation methods are only trained on a single frame of an image, and the correlation between frames is not considered, which leads to unstable segmentation on continuous frame image sequences. A few methods consider the correlation of video sequences, such as methods based on key frames. These methods may cause the cumulative error of semantic segmentation to become increasingly serious as the distance between the current frame and the key frame increases.

In this work, we proposed a video lanes and road markers segmentation algorithm, which includes image sequence processing and an end-to-end trainable multiple-input single-output network to jointly handle lane and road marker segmentation. Our method involves estimating the optical flow of adjacent frames, and then using the segmentation of the previous frame to crop the target area of the past frame and its neighborhood through a mask, thereby emphasizing the location of high-probability targets in adjacent frames, and providing region proposal for the semantic segmentation network, guiding the network to move its attention to the area near the target in the past frame. Finally, the optimized network uses optical flow information to refine the segmentation results. Tests on the Apolloscape lanemark segmentation dataset and the CULane dataset showed that compared with single-frame image segmentation algorithms and other semantic video segmentation algorithms, our method achieves higher accuracy in image sequences, and acheived improvements in multiple evaluations. Especially for targets such as turning arrows, broken lines, zebra crossings, and opposite lanes blocked by railings, as well as in dazzle light, arrows, and night scenes, our method provides significant accuracy improvements. Among the robust methods, the segmentation speed of video lane and road marker is faster by 2.5 times and accuracy is higher by 1.4%. In the worst case, the time consistency is reduced by only 2.2% at most. In future work, we will continue to study the segmentation and detection of lanes and road markers. Our goal is to explore novel pipelines that use continuous frame information to further improve the segmentation accuracy and stability of target edges in image sequences.

## Figures and Tables

**Table 1:** The mIoU classes, mIoU categories, and TC of our model under two training strategies.

Training Strategy	mIoU cls	mIoU cat	TC
Direct joint training	0.708	0.891	0.719
Two-stage training	**0.776**	**0.953**	**0.771**

**Table 2:** The mIoU classes, mIoU categories, and TC of our model with dilation kernels of different sizes.

Clipping-Net Branch		mIoU cls	mIoU cat	TC
η	μ			
0.1	1	0.693	0.877	0.632
0.1	8	0.699	0.879	0.678
0.5	8	0.713	0.892	0.704
0.5	25	0.769	0.946	0.763
1.2	25	**0.776**	**0.953**	**0.771**
1.2	50	0.773	0.951	0.770
2.3	50	0.768	0.947	0.770
2.8	50	0.767	0.945	0.769
2.8	80	0.754	0.928	0.761
Full image (without cropping)		0.742	0.910	0.728

**Table 3:** The mIoU of our model that removes the Clipping-net branch and our model that contains the Clipping-net branch in each class.

Category	Class	No Clipping-Net Branch	Clipping-Net Branch	
			η=1.2 and μ=25	η=2.8 and μ=50
Dividing	White solid	0.995	0.997	0.987
	Yellow solid	0.871	0.903	0.873
	Yellow double solid	0.903	0.909	0.895
	White solid and broken	0.863	0.894	0.887
Guiding	White broken	0.705	0.715	0.706
	Yellow broken	0.847	0.878	0.851
Stopping	White solid	0.789	0.792	0.787
Parking	White solid	0.667	0.679	0.662
Zebra	Crosswalk	0.858	0.921	0.854
Rotation arrow	White thru	0.653	0.712	0.681
	White thru and left	0.668	0.698	0.663
	White thru and right	0.732	0.768	0.758
	White left	0.730	0.744	0.741
	White right	0.619	0.626	0.620
	White left and right	0.566	0.587	0.541
Reduction	Speed bump	0.571	0.617	0.579
Attention	Zebra attention	0.743	0.759	0.733
No park	No parking	0.759	0.761	0.752
Average		0.752	**0.776**	0.754

**Table 4:** Segmentation score for lanes and road markers on the Apolloscape lanemark segmentation dataset by different methods.

Method		mIoU cls	mIoU cat	TC	fps
Single frame	VPGNet [18]	0.720	0.905	0.653	42
	DeepLabv3+ [39]	0.761	0.942	0.706	21
	PSPNet [38]	0.748	0.921	0.683	30
	Dilation-CNN [40]	0.673	0.859	0.611	59
	HRNet [41]	0.735	0.916	0.664	87
Multiframe	PSPNet + GRFP [22]	0.755	0.935	0.761	26
	PSPNet + NetWarp [24]	0.754	0.931	0.767	25
	PSPNet + Eff. [26]	0.762	0.938	**0.793**	30
	Dilation + GRFP [22]	0.687	0.864	0.679	54
	Dilation + NetWarp [24]	0.688	0.863	0.692	51
	HRNet + Eff. [26]	0.744	0.923	0.785	87
	**Ours**	**0.776**	**0.953**	0.771	74

**Table 5:** Comparison of different methods on CULane, with an IoU threshold of 0.5. For crossroad, only FPs are shown.

Category	SCNN [16]	ENet-SAD [42]	LaneATT [43]			CondLaneNet [31]			Ours
			Small	Medium	Large	Small	Medium	Large	
Normal	90.60	90.10	91.17	92.14	91.74	92.87	93.38	**93.47**	92.69
Crowded	69.70	68.80	72.71	75.03	76.16	75.79	77.14	**77.44**	76.37
Dazzle light	58.50	60.20	65.82	66.47	69.74	70.72	71.17	70.93	**71.34**
Shadow	66.90	65.90	68.03	78.15	76.31	80.01	79.93	**80.91**	80.52
No line	43.40	41.60	49.13	49.39	50.46	52.39	51.85	**54.13**	51.71
Arrow	84.10	84.00	87.82	88.38	86.29	89.37	89.89	90.16	**90.19**
Curve	64.40	65.70	63.75	67.72	64.05	72.40	73.88	**75.21**	71.45
Night	66.10	66.00	68.58	70.72	70.81	73.23	73.92	74.80	**74.93**
Crossroad	1990	1998	**1020**	1330	1264	1364	1387	1201	1376
Total	71.60	70.80	75.13	76.68	77.02	78.14	78.74	**79.48**	78.32
TC	0.634	0.667	0.691	0.705	0.708	0.732	0.737	0.749	**0.783**
FPS	17.2	82	**277**	197	30	248	171	65	89

## Data Availability

Publicly available datasets were analyzed in this study. These data can be found here: Apolloscape Lanemark Segmentation dataset (http://apolloscape.auto/lane_segmentation.html, accessed on 28 October 2021), CULane Dataset (https://xingangpan.github.io/projects/CULane.html, accessed on 28 October 2021).
